# A Yeast-Based Chemical Screen Identifies a PDE Inhibitor That Elevates Steroidogenesis in Mouse Leydig Cells via PDE8 and PDE4 Inhibition

**DOI:** 10.1371/journal.pone.0071279

**Published:** 2013-08-14

**Authors:** Didem Demirbas, Arlene R. Wyman, Masami Shimizu-Albergine, Ozgur Cakici, Joseph A. Beavo, Charles S. Hoffman

**Affiliations:** 1 Biology Department, Boston College, Chestnut Hill, Massachusetts, United States of America; 2 Department of Pharmacology, University of Washington, Seattle, Washington, United States of America; University of Calgary, Canada

## Abstract

A cell-based high-throughput screen (HTS) was developed to detect phosphodiesterase 8 (PDE8) and PDE4/8 combination inhibitors. By replacing the *Schizosaccharomyces pombe* PDE gene with the murine PDE8A1 gene in strains lacking adenylyl cyclase, we generated strains whose protein kinase A (PKA)-stimulated growth in 5-fluoro orotic acid (5FOA) medium reflects PDE8 activity. From our previously-identified PDE4 and PDE7 inhibitors, we identified a PDE4/8 inhibitor that allowed us to optimize screening conditions. Of 222,711 compounds screened, ∼0.2% displayed composite Z scores of >20. Additional yeast-based assays using the most effective 367 compounds identified 30 candidates for further characterization. Among these, compound BC8-15 displayed the lowest IC_50_ value for both PDE4 and PDE8 inhibition in *in vitro* enzyme assays. This compound also displays significant activity against PDE10A and PDE11A. BC8-15 elevates steroidogenesis in mouse Leydig cells as a single pharmacological agent. Assays using BC8-15 and two structural derivatives support a model in which PDE8 is a primary regulator of testosterone production by Leydig cells, with an additional role for PDE4 in this process. BC8-15, BC8-15A, and BC8-15C, which are commercially available compounds, display distinct patterns of activity against PDE4, PDE8, PDE10A, and PDE11A, representing a chemical toolkit that could be used to examine the biological roles of these enzymes in cell culture systems.

## Introduction

The second messengers cyclic adenosine monophosphate (cAMP) and cyclic guanosine monophosphate (cGMP) mediate a diverse range of cellular processes [1]. Control of cyclic nucleotide signals in mammalian cells is tailored, in part, by biochemically-distinct phosphodiesterases (PDEs) that hydrolyze cAMP and/or cGMP to 5′ adenosine monophosphate and 5′ guanosine monophosphate, respectively. Due to their importance in governing the amount and spatiotemporal distribution of cyclic nucleotides, PDEs are considered important drug targets. The PDE superfamily consists of 11 families, classified according to their structure, regulation, biochemical and pharmacological characteristics [1]. Among these, PDE4, PDE7 and PDE8 enzymes are cAMP-specific PDEs. Rolipram is a PDE4-selective inhibitor often used to define the PDE4 family. PDE4-selective inhibitors have anti-inflammatory, anti-depressant, and pro-cognitive effects [1], [2]. PDE7 is also implicated in inflammation, however less is known about PDE7 function, partially due to the fact that selective inhibitors have only recently been described [3]–[5]. PDE7 inhibitors BRL50481 and BC30 enhance the reduction in TNFα secretion from stimulated U937 cells conferred by rolipram-mediated PDE4 inhibition [3].

PDE8 enzymes, encoded by the PDE8A and PDE8B genes, display high affinity and specificity for cAMP [6]. Studies using PDE8A and PDE8B knock-out mice have identified important functions of the PDE8 family in steroidogenesis. Leydig cells from PDE8A knock-out mice show increased sensitivity to lutenizing hormone (LH) for testosterone production [7]. PDE8A is also important in other processes such as T cell activation, effector T cell adhesion and excitation-contraction coupling in ventricular myocytes [8], [9]. Studies of PDE8B knock-out mice, along with pharmacological evidence, indicate that PDE8B is a major regulator of adrenal steroidogenesis [10]. Until recently, chemical approaches to studying PDE8 function have been hampered by the fact that PDE8 enzymes are insensitive to the general PDE inhibitor 3-isobutyl-1-methylxanthine (IBMX) and are only weakly inhibited by dipyridamole [6], [11]. In 2010, Pfizer reported a potent and selective PDE8 inhibitor, PF-04957325, that is starting to be used to study PDE8 function [8], [10], [12]. At the same time, gene association studies are beginning to shed light on roles for PDE8A and PDE8B, such as in HIV-1 replication [13] and adrenal function [14].

Just as we observed a synergistic effect on TNFα release by macrophage in response to inhibition of both PDE4 and PDE7 enzymes [3], it has been shown that inhibition of both PDE4 and PDE8 enzymes produces a significant elevation of testosterone production by Leydig cells [12]. Thus, availability of structurally-diverse PDE4/8 dual inhibitors will be useful to the study of function of both PDE families.

Here, we describe the adaptation and deployment of a fission yeast-based screening platform to detect PDE8 and dual PDE4/8 inhibitors, leading to the identification of a PDE4/8 inhibitor that elevates steroidogenesis in mouse Leydig cells. *Schizosaccharomyces pombe* monitors extracellular glucose levels through a cAMP signaling pathway [15]. Most of the components of the glucose/cAMP pathway were identified in a genetic screen that utilizes a fusion of the *ura4* ^+^ gene of the uracil biosynthetic pathway to the PKA-repressed *fbp1* ^+^ gene [16]. Recently, we adapted the use of this reporter to monitor heterologously-expressed mammalian PDEs [3], [17]–[19]. Low PKA activity allows *fbp1-ura4* expression that confers sensitivity to the pyrimidine analog 5-fluoro orotic acid (5FOA). Pharmacological inhibition of heterologously-expressed PDEs allows PKA-mediated repression of *fbp1-ura4* to confer 5FOA-resistant (5FOA^R^) growth, which is detected in HTSs using 384-well plates. We optimized screening conditions to conduct a 222,711 compound HTS to discover a potent PDE4/8 inhibitor. As with our prior PDE4 and PDE7 studies, compounds identified in this screen show cell permeability and biological activity in mammalian cell culture assays without further development via medicinal chemistry. Here, we describe BC8-15, a PDE inhibitor that elevates steroid production by mouse Leydig cells. Our data suggest that the ability to inhibit both PDE4 and PDE8 is key to the biological activity of BC8-15. In addition, two derivatives of BC8-15 display different profiles of activity against PDE4, PDE8, PDE10, and PDE11 to create a chemical toolkit that could be used to study the relative roles of these enzymes in various biological assays.

## Materials and Methods

### Ethics Statement

All procedures involving mice were reviewed and approved by the Institutional Animal Care and Use Committee of the University of Washington, and were carried out in accordance with the National Institutes of Health *Guide for Care and Use of Laboratory Animals*. Animals were euthanized by an overdose of pentobarbital to ameliorate suffering prior to removal of testes and Leydig cell isolation.

### Yeast Strains, Media, and Growth Conditions

The genotypes of yeast strains used in this study are presented in Table 1. Cells were grown at 30°C in YEA-rich, EMM-defined, or 5FOA media as previously described [16], [20], [21]. Insertions of the murine PDE8A1 gene or the human PDE4A1, PDE7A1 or PDE7B1 gene into the *S. pombe cgs2* (PDE) locus have been previously described (the *cgs2* PDE gene was chosen as the site of integration so as to express the mammalian PDEs at a physiologically-relevant level in our strains) [18]. A plasmid expressing the catalytic domain of murine PDE8A was constructed by PCR-amplifying and cloning a 1325 bp region of the PDE8A gene (codons 443 to 824 of PDE8A1) into *EcoRI*-linearized pNMT1 (Invitrogen, Carlsbad, CA) vector via gap repair transformation [22]. DNA sequencing confirmed that the plasmid carried the wild type sequence of the PDE8A catalytic domain. The resulting plasmid was linearized by digestion with *Blp*I and integrated into the *ars1* locus on *S. pombe* chromosome 1 to create a strain that stably expresses the PDE8A catalytic domain (DDP40: Table 1).

**Table 1:** Strain list.

Strain	Genotype						
CHP1189	*h* ^+^	*fbp1-ura4^+^*	*ura4::fbp1-lacZ*	*leu1-32*	*gpa2*Δ	*pap1*Δ	*cgs2*Δ::PDE7A1
CHP1204	*h* ^−^	*fbp1-ura4^+^*	*ura4::fbp1-lacZ*	*leu1-32*	*git2*Δ	*pap1*Δ	*cgs2*Δ::PDE8A1
CHP1209	*h* ^−^	*fbp1-ura4^+^*	*ura4::fbp1-lacZ*	*leu1-32*	*git3*Δ	*pap1*Δ	*cgs2*Δ::PDE7B1
CHP1262	*h* ^−^	*fbp1-ura4^+^*	*ura4::fbp1-lacZ*	*leu1-32*	*gpa2*Δ	*pap1*Δ	*cgs2*Δ::PDE4A1
DDP40	*h* ^+^	*fbp1-ura4^+^*	*ura4::fbp1-lacZ*	*leu1-32*	*cgs2-2*	*pap1*Δ	*ars1*::pNMT1-PDE8Acat

### Small Molecules

Small molecules were purchased from ChemDiv (San Diego, CA; BC8-15: E973-0331, BC8-15A: E881-0067, BC8-15C: C301-5319).

### 5FOA Growth Assays

Cyclic nucleotide-dependent growth in 5FOA medium was monitored in 50 µL cultures in 384-well microtiter plates (untreated, flat clear bottom). Cells were inoculated at an initial cell density of 0.5–2×10^5^ cells/mL. Previously described optimized pre-growth conditions for each strain were used to repress the *fbp1-ura4^+^* reporter prior to inoculation into 5FOA medium, as cells that are already expressing the Ura4 enzyme would be 5FOA-sensitive even upon exposure to a PDE inhibitor [18]. Following a 48-h incubation at 30°C, plates were vortexed and optical densities (600 nm) were measured. The experiments with the PDE8A-expressing strain (CHP1204) were performed with 0.5 mM cAMP in the pre-growth medium and 40 µM cAMP in the 5FOA medium. For the strain that expresses the catalytic domain of PDE8A (DDP40), the initial cell density in 5FOA medium was 1×10^5^ cells/mL. EMM medium containing 20 µM BC69 was used to culture DDP40 cells prior to transferring cells to 5FOA medium (as DDP40 cells express adenylyl cyclase, no cAMP was added to the 5FOA medium, and a PDE8 inhibitor is used to repress *fbp1-ura4* reporter transcription prior to exposure of cells to 5FOA). Dose response profiling of compounds (0.1 µM to 66.6 µM) was performed in 5FOA medium.

### High-throughput Screens (HTSs)

The suitability and quality of the assay conditions for high throughput screening was evaluated by a Z factor test as previously described [19], [23], using DMSO (0.2%) as a negative control, and either 5 mM cAMP, 10 µM BC69, or 20 µM BC69 as positive controls. HTSs were performed at the Harvard Medical School ICCB Screening Facility. 100 nL compounds were pinned from stock solutions of 5 mg/mL into wells of 384-well microtiter dishes containing 25 µL 5FOA medium. Exponential phase CHP1204 cells were then transferred into the wells to a final density of 0.5×10^5^ cells/mL. The 5FOA medium contained 40 µM cAMP as CHP1204 cells lack adenylyl cyclase and require exogenous cAMP to activate PKA. Finally, each screening plate included four wells containing 0.2% DMSO in the medium and four wells containing 20 µM BC69 in the medium as in-plate negative and positive controls. After 48 h growth at 30°C, the optical density (OD_600_) of each well was measured using a plate reader. Composite Z scores were calculated for each compound by scaling the vector [Zscore_A, Zscore_B] by the cosine correlation with the diagonal (i.e. identical Z-scores in both replicates).

### *In vitro* PDE Assays

*In vitro* PDE assays were performed as previously described [24], using the Ba(OH)_2_ precipitation assay [25], with recombinant human PDE1C, PDE3B, PDE5A1, PDE8A, PDE9A2, PDE10A1, PDE11A4 (BPS Bioscience Inc.), rat PDE2A (BPS Bioscience Inc.), human PDE7A (BIOMOL International), and human PDE4A10 enzymes (gift from Dr. Hengming Ke). Substrate concentrations were 120 nM cGMP (PDE1C), 1 µM cGMP (PDE2A), 30 nM cGMP (PDE3B), 50 nM cAMP (PDE4A), 520 nM cGMP (PDE5A), 10 nM cAMP (PDE7A), 10 nM cAMP (PDE8A), 70 nM cGMP (PDE9A), 40 nM cAMP (PDE10A), and 120 nM cGMP (PDE11A). Reactions were performed in 50 mM Tris·HCl (pH 8.5), 10 mM MgCl_2_ (for all enzymes except PDE8) or 4 mM MnCl_2_ (for PDE8A), and 5 mM dithiothreitol, and proceeded for 30 min for PDE8A, 6 min for PDE10A and 15 min for other PDEs, so that reactions were linear with time. Reactions were stopped with 200 µL 0.2 M ZnSO_4_, after which 5′-AMP was precipitated with 200 µL 0.25 N Ba(OH)_2_ by centrifugation for 15 min at 14000 rpm. The remaining radioactivity in the supernatant was measured in 4 mL of scintillation fluid. The amount of hydrolysis was generally limited to 30 percent conversion and IC_50_ values were calculated as the means of three independent experiments. Substrate concentrations were ≤0.1*Km for each enzyme, thus IC_50_ values approximate the Ki values.

### Measurement of Testosterone Production from Primary Leydig Cells

Leydig cells were isolated from testes of wild type and PDE8A^−/−/^B^−/−^ knock-out adult mice as previously described [7]. Digested tissue from decapsulated mouse testis was enriched for interstitial cells by filtration as previously described [12]. Collected cells were resuspended in DMEM/F-12 medium supplemented with 1 mg/mL BSA and 150 µL aliquots were dispensed into 48-well plates. After permitting cells to recover at 37°C for 3 h, 30 µL of media with test compounds or vehicle was added to replicate wells. Media were collected after incubation for 3 h and stored at −20°C. Testosterone released into the media was measured by a testosterone ELISA kit (Neogen, Lexington, KY).

### Measurement of Steroid Release from Mouse Leydig Tumor Cells

MA-10 Leydig tumor cells were maintained in RPMI medium supplemented with 10% horse serum and seeded in 24 well plates to achieve 60–80% confluency after 2–3 days. Cells were washed with serum free medium twice and incubated in a 5% CO_2_ incubator at 37°C for 2 h. Following incubation, cells were treated with BC8-15, BC8-15A, or BC8-15C by replacing the media with 300 µL RPMI medium containing test compounds or vehicle. Media were collected after incubation for 2 h at 37°C. Progesterone levels in the media were measured using a progesterone ELISA kit (Neogen, Lexington, KY). Protein levels were determined by lysing cells in lysis buffer (50 mM Tris HCl (pH 7.5), 150 mM NaCl, 1% Triton X-100, 2 mM PMSF (supplemented with a Complete Protease Inhibitor Tablet (Roche, Nutley, NJ)) and measuring the protein concentration using a BCA protein assay kit (Pierce, Rockford, IL).

### Docking Simulation of BC8-15 on PDE4A and PDE8A Structures

The docking simulations of BC8-15 with PDE8A (PDB ID: 3ECN) and PDE4A (PDB ID: 2QYK) were carried out using AutoDock4 software through the AutoDockTools GUI [26]. Hydrogen atoms were added to all molecules at predicted positions and Gasteiger charges were calculated by the AutoDock ADT tool. During the docking calculations, the complex structure was treated as a rigid molecule. The three dimensional structure of BC8-15 was created from its SMILES using Online SMILES Translator and Structure File Generator (National Cancer Institute/CADD Group). As the docking site, the reaction groove on the surface of the PDE structures was chosen and grid box was built with the default grid spacing (0.375 Å). Lamarckian genetic algorithm was executed for the docking simulation with the following parameters: 50 runs, 150 population, 2,500,000 evaluations, and 27,000 generations. Default values were used for all other parameters. The calculated binding orientations of the BC8-15 were ranked according to interaction energy. Among the solutions, the final conformation of the docked ligand was chosen based on its orientation, calculated interaction energy, and RMSD from the reference (starting) conformation.

## Results

### Development of a Fission Yeast-based HTS for PDE8 Inhibitors

**Figure 1:**
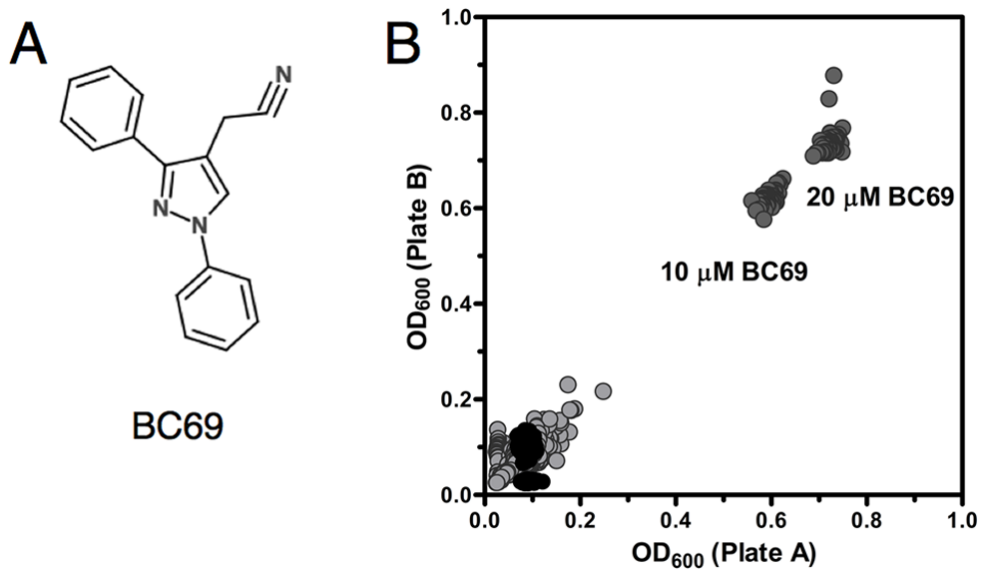
Development of a PDE8A inhibitor HTS. **A)** Structure of PDE4/8 inhibitor BC69 used to optimize PDE8 inhibitor assay. **B)** A yeast-based screen finds no potent PDE8 inhibitors in a Known Bioactives Collection. PDE8A-expressing strain CHP1204 was screened against 2,640 known bioactive compounds in duplicate wells (plate A and B). Scatter plot of absorbance values from plate A against plate B is shown. Negative control wells contained 0.2% DMSO (black circles), positive control wells contained 10 or 20 µM BC69 (dark gray circles). Experimental wells are shown in light gray. The data set has a correlation value of 0.996.

We previously carried out HTSs for PDE4, PDE7, and PDE11 inhibitors utilizing strains of the fission yeast *Schizosaccharomyces pombe* whose protein kinase A (PKA) activity reflects the activity of heterologously-expressed mammalian PDEs [3], [19], [24]. In PDE4- and PDE7-expressing strains, inhibition of PDE activity elevated endogenously-produced cAMP levels to activate PKA, which in turn repressed transcription of a *fbp1-ura4* reporter, allowing cell growth in 5FOA medium [3], [19]. The PDE11A-expressing strain lacked adenylyl cyclase, so that PKA activity was controlled by the addition of cGMP to the growth medium [24]. As murine PDE8A1 displays relatively low cAMP-hydrolyzing activity in our strains, we adopted a strategy similar to that of the PDE11A inhibitor screen using strain CHP1204 that lacks adenylyl cyclase, so that PKA activity is controlled by the addition of cAMP to the growth medium. 40µM cAMP permits 5FOA^R^ growth of a strain lacking PDE activity, but not of strain CHP1204; thus under these conditions PDE8A inhibition results in 5FOA^R^ growth. By screening 56 compounds obtained from our previous PDE4 and PDE7 HTSs, we identified a PDE4/8 dual selective inhibitor (BC69; Figure 1A) [17] that was used to optimize screening conditions for a PDE8 inhibitor HTS.

### Screening of Known Bioactive Compounds

Strain CHP1204 was used to screen 2,640 compounds from the Known Bioactives Collection of the Harvard Medical School ICCB Screening Facility (BIOMOL 2 ICCB Known Bioactives- High Conc., Prestwick1 Collection and NINDS Custom Collection 2). The screening conditions yielded Z factor values of 0.8 for 10 µM BC69 and 0.83 for 20 µM BC69 positive controls, and high reproducibility between the duplicate plates (R = 0.996). Two compounds from the Known Bioactives Collection weakly promoted 5FOA^R^ growth of the PDE8A1-expressing strain with composite Z scores of 12.9 and 10.1 (Figure 1B). The most effective compound was the steroid epiandrosterone, a natural metabolite of dehydroepiandrosterone. However, 32 µM epiandrosterone inhibited recombinant human PDE8A by only 17% in an *in vitro* PDE assay (data not shown). The absence of effective PDE8 inhibitors among the known bioactive compounds was not surprising as no potent PDE8 inhibitor had been described in the literature prior to compound PF-04957325 [8].

### Screening Commercial Libraries

The screen for PDE8A inhibitors was performed using 220,071 compounds from commercial libraries maintained at the Harvard Medical School ICCB Screening facility (Asinex 1, Biomol-TimTec1, Bionet 1, Bionet 2, CEREP, ChemBridge 3, ChemDiv 1, ChemDiv 2, ChemDiv 3, ChemDiv 4, ChemDiv 6, Enamine 1, Enamine 2, I.F. Lab 2, Life Chemicals 1, MayBridge 1, MayBridge 2, MayBridge 3, Maybridge 4, Maybridge 5, MixCommercial Plate 1, MixCommercial Plate 2, MixCommercial Plate 3, Mix Commercial Plate 5, and Peakdale 1). Positive hits were identified according to the average optical density (OD) values of duplicate wells and the ability of compounds to produce composite Z scores of >8.53, a previously-described cutoff value for HTSs [27]. A total of 2,266 hits (1.03%) were identified, of which 1,815 produced OD values between 0.2 and 0.4, 388 produced OD values between 0.4 and 0.6, and 63 produced OD values above 0.6. Table 2 summarizes the HTS with regard to the number of compounds falling within composite Z score intervals.

**Table 2:** Summary of the HTS data.

Composite Z Score	Number of compounds
−12 to −8	377
−8 to −4	2,920
−4 to 0	114,020
0 to 4	95,325
4 to 8	7,203
8 to 12	1,475
12 to 16	554
16 to 20	313
>20	524

The ability of 220,071 small molecules to promote 5FOA^R^ growth of a PDE8A-expressing strain was assessed by their composite Z scores (see Materials and Methods). The number of compounds within each indicated composite Z score interval is presented.

The most effective 367 candidate compounds were retested against strain CHP1204 (PDE8A1) as well as strains that express human PDE4A1, human PDE7A1 or human PDE7B1, and the catalytic domain of murine PDE8A. Thirty compounds, based on either potency or selectivity, were acquired for further testing. Eleven of these compounds inhibited recombinant PDE8A with IC_50_ values of ≤10 µM in *in vitro* enzyme assays. All PDE8A inhibitors displayed similar potency in enzyme assays using recombinant human PDE8B (data not shown). Here, we describe BC8-15 that displays the lowest IC_50_ value for both PDE4A and PDE8A among these compounds.

### Specificity Profiling of BC8-15 and Related Compounds

**Figure 2:**
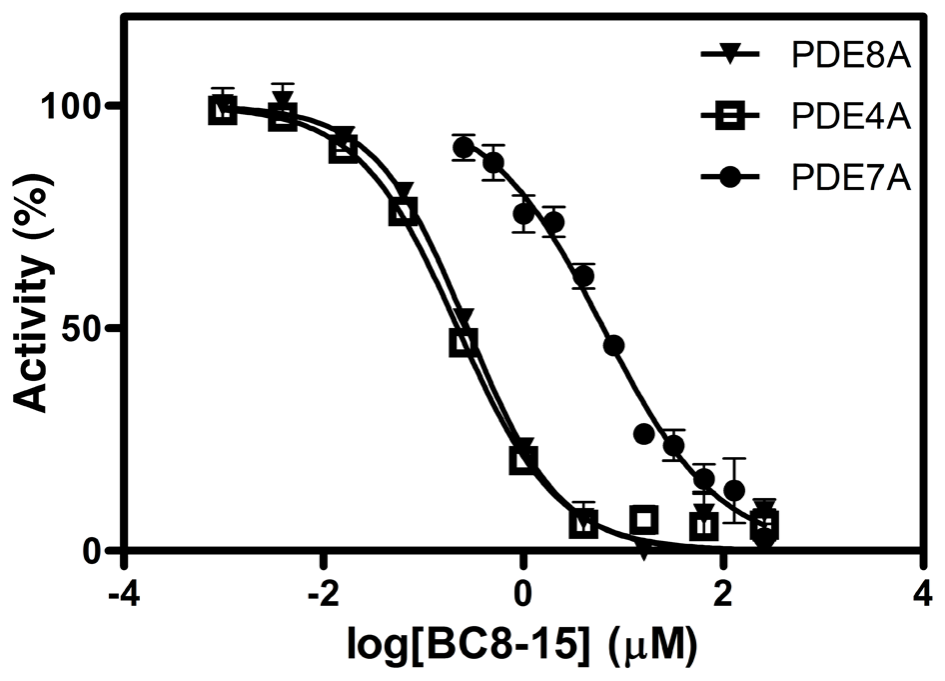
Inhibition of PDE4A, PDE7A and PDE8A by BC8-15 as determined by *in vitro* enzyme assays. BC8-15 inhibitory activity was measured by *in vitro* PDE assays as described in Materials and Methods. Substrate concentrations are 10 nM cAMP for PDE8A and PDE7A and 50 nM cAMP for PDE4A. Each assay was performed at 10 different compound concentrations in duplicate reaction tubes. IC_50_± SD values are determined by performing non-linear regression analysis on three independent experiments.

**Figure 3:**
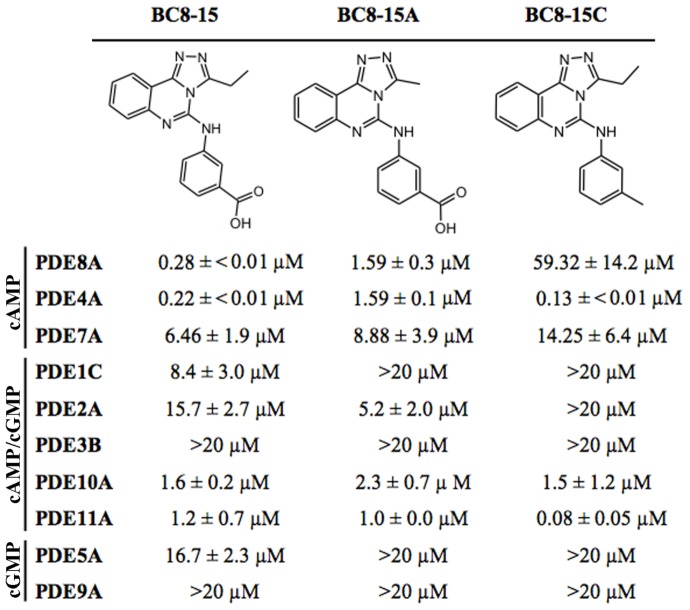
IC_50_ values for BC8-15 and related compounds. The structures of BC8-15 and two structurally-related derivatives are shown. The IC_50_ values of each compound were determined by *in vitro* PDE assays (see Material and Methods). The values represent mean IC_50_± SD determined from three independent experiments.

While several compounds conferred greater 5FOA^R^ growth to PDE8A-expressing strains, BC8-15 was the most potent PDE8A inhibitor based on *in vitro* enzyme assays. BC8-15 inhibits PDE8A and PDE4A with IC_50_ values of 280 and 220 nM, respectively, while showing 23-fold less activity against PDE7A (IC_50_ = 6.46µM) (Figure 2), a third member of the family of cAMP-specific PDEs. *In vitro* enzyme assays characterizing BC8-15 derivatives identified two useful analogs. BC8-15A displays reduced potency against both PDE4 and PDE8; BC8-15C displays severely reduced potency against PDE8 while retaining activity against PDE4 (Figure 3). As mentioned above, these compounds show similar activity against PDE8B as against PDE8A (data not shown). Further *in vitro* enzyme assays characterizing BC8-15, BC8-15A, and BC8-15C against PDEs from the PDE1, PDE2, PDE3, PDE5, PDE9, PDE10, and PDE11 families show that all three compounds are moderately effective PDE10A and PDE11A inhibitors (Figure 3), with the exception that BC8-15C is a potent PDE11A inhibitor.

### BC8-15 Elevates Steroidogenesis in Mouse Leydig Cells via PDE8 and PDE4 Inhibition

**Figure 4:**
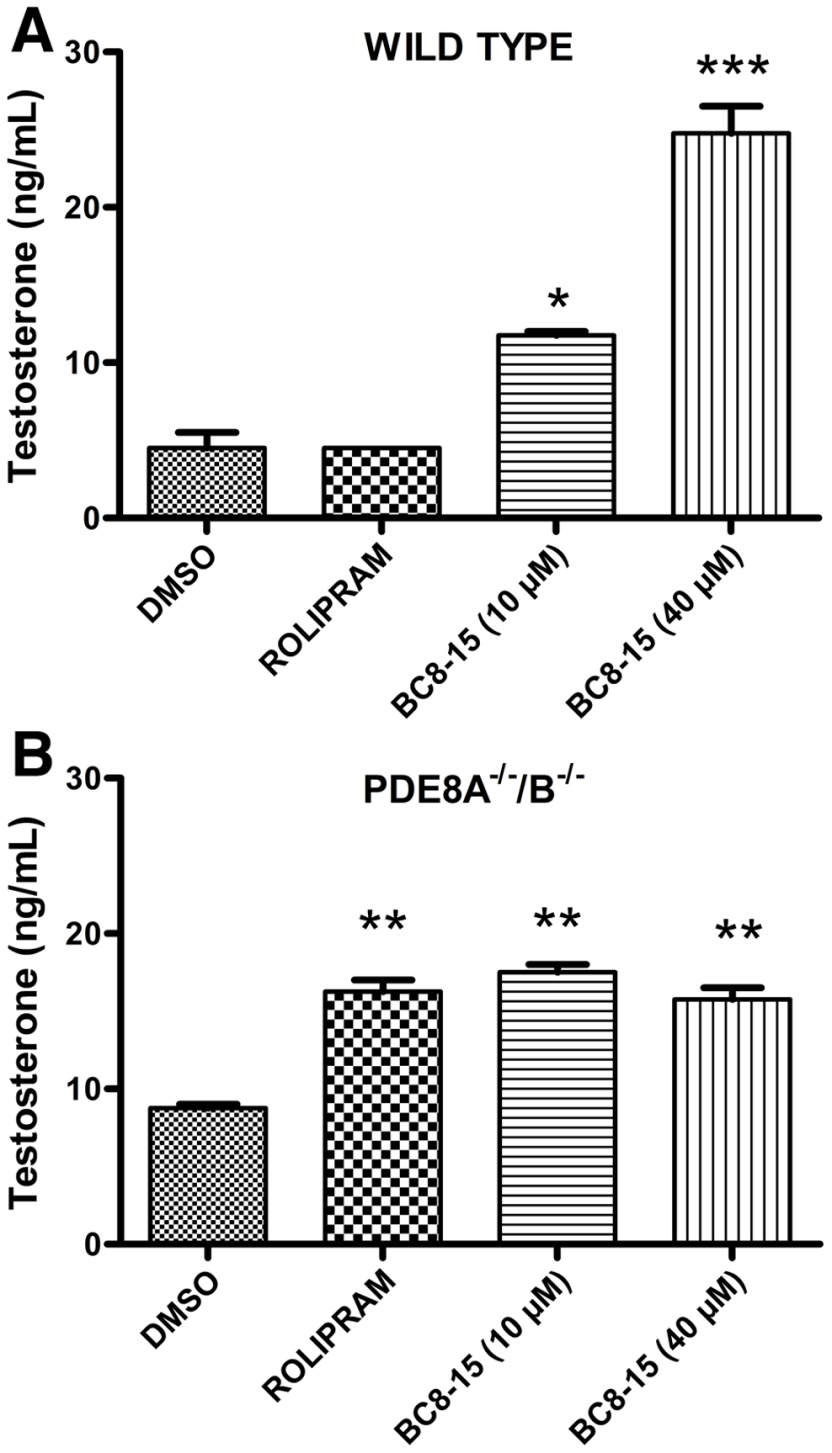
BC8-15 elevates testosterone release by primary Leydig cells. Leydig cells isolated from wild-type (Panel A) and PDE8A^−/−/^B^−/−^ knock-out (Panel B) mice were treated with rolipram (20 µM) or BC8-15 (10 µM and 40 µM) for three hours. Testosterone released into the media was assayed in duplicate for each condition. Values represent the mean ± SEM. Data from wild type and knock-out samples were separately analyzed with one-way ANOVA followed by Tukey’s multiple comparison test. Significant differences are indicated in comparison to the DMSO control (* p<0.05, ** p<0.01, *** p<0.001).

The effect of BC8-15 on testosterone production was examined using primary Leydig cells isolated from the testes of either wild type or PDE8A^−/−/^B^−/−^ knock-out adult mice [12]. Following isolation and incubation to allow cell attachment, cells were treated with compounds at 10 µM and 40 µM for three hours (Figure 4). Leydig cells from wild type mice show no response to PDE4 inhibition by rolipram, but produce a dose-dependent elevation in testosterone release in response to BC8-15. This was expected based on the effect of individual PDE4 and PDE8 inhibitors on testosterone release [12]. Basal testosterone release by PDE8A^−/−/^B^−/−^ Leydig cells is approximately twice that of wild type levels, consistent with PDE8 playing an important role in controlling testosterone production. Unlike wild type cells, PDE8A^−/−/^B^−/−^ cells respond to both rolipram and BC8-15 with a two-fold increase in testosterone production (Figure 4). Furthermore, as BC8-15 produces a similar elevation to that of rolipram on PDE8A^−/−/^B^−/−^ cells, PDE10A and/or PDE11A do not appear to regulate steroidogenesis, as both are inhibited by BC8-15, but not by rolipram.

**Figure 5:**
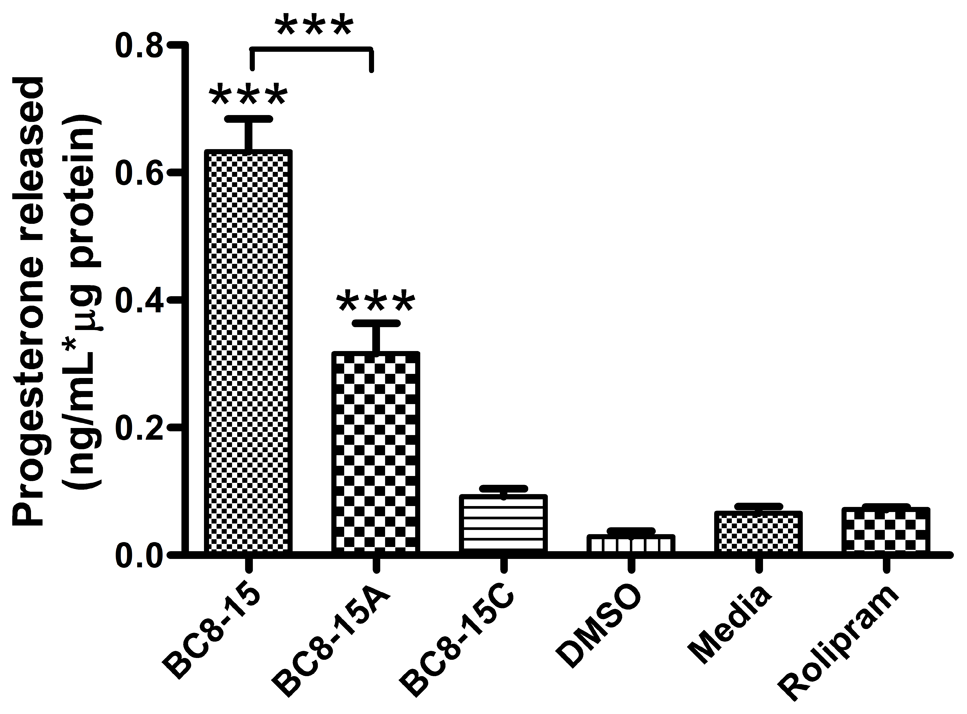
BC8-15 derivative elevation of progesterone release by MA-10 Leydig tumor cells corresponds with PDE8 inhibitory activity. MA-10 cells were treated with 20 µM of rolipram, BC8-15, BC8-15A or BC8-15C. Progesterone released into the media after 2 h was measured using a progesterone ELISA kit (Neogen). The data are representative of three independent experiments with 2–4 sample wells for each condition. Values are the mean of four experimental wells ± SEM. Data were analyzed with one-way ANOVA followed by a Tukey’s multiple comparison test (*** p<0.001). The response to BC8-15 is significantly different from that for BC8-15A (p<0.001) and both are significantly different from all other groups (p<0.001).

The effect of BC8-15 on steroidogenesis by Leydig cells was also tested by monitoring progesterone production from Leydig tumor MA-10 cells. As these cells have undetectable 17 alpha-hydroxylase activity, their major steroid output is progesterone [28]. BC8-15 increased progesterone release by >10-fold compared to the untreated cells, while DMSO and rolipram had little or no effect (Figure 5). Exposure to BC8-15A, which displays reduced activity against both PDE4 and PDE8 (Figure 3), increased progesterone release five-fold, while BC8-15C, which only displays activity against PDE4 (Figure 3), had little or no effect on progesterone release (Figure 5). While the ability of these compounds to elevate progesterone release corresponds to their PDE8 inhibitory activity, their effectiveness on MA-10 cells is likely to be co-dependent on their PDE4 inhibitory activity. These results are consistent with the hypothesis that the effect of BC8-15 on basal steroid production by wild type Leydig cells is due to PDE8 inhibition and PDE4 co-inhibition, and not due to PDE4 inhibition alone, PDE10A or PDE11A inhibition, or to an off-target activity.

## Discussion

Studies on PDE8 function are beginning to uncover roles in steroidogenesis in both Leydig cells and the adrenal gland, as well as roles in T cell activation, migration of activated lymphocytes, effector T cell adhesion and excitation-contraction coupling in ventricular myocytes [7]–[10], [12], [29], [30]. However, such studies have been impeded by the lack availability of effective PDE8 inhibitors until the recent report of Pfizer’s PF-04957325. To discover PDE4/8 inhibitors as well as novel PDE8 inhibitors, we optimized and conducted a 222,711 compound HTS based on PKA-regulated growth of an *S. pombe* strain that expresses murine PDE8A1 as its only PDE, with follow-up screens of candidate compounds against PDE4-expressing strains. This led to the identification of BC8-15, which potently elevates steroidogenesis when used alone in mouse Leydig cells.

A screen of known bioactive compounds did not identify an effective PDE8 inhibitor. Although dipyridamole, which non-selectively inhibits PDE8 [6], was in the collection, it showed no activity in this screen. This appears to be due to poor solubility of dipyridamole in the yeast medium or its inability to be taken up by yeast, as we have not detected a growth response to dipyridamole with either PDE5A- or PDE8A-expressing strains (data not shown). From the bioactive compound collection, epiandrosterone gave the highest composite Z score- indicating a statistically-significant response relative to negative control wells; although the increase in optical density was modest. While it is tempting to speculate that epiandrosterone, a metabolite of dehydroepiandrosterone, could be a natural regulator in steroidogenesis, it only weakly inhibits recombinant PDE8A in an *in vitro* enzyme assay. While we had also identified steroids (androsterone acetate and canrenone) in our PDE7 inhibitor screen [3], in this case they were among our most effective hit compounds in contrast to the weak effect of epiandrosterone in this current study.

The HTS against other commercial libraries identified 2,266 compounds that promoted growth of the PDE8A-expressing strain to a statistically-significant level relative to DMSO-pinned controls. Fifty-six of the 63 strong hits that produced OD values of >0.6 came from only six libraries (ChemBridge 3, ChemDiv 3, ChemDiv 4, Enamine 1, Enamine 2, and MayBridge 3) that represented 37% of the screened compounds. These libraries had a hit rate of 0.07%, in comparison to 0.005% for the other 19 libraries. Examination of hit compounds revealed few structurally-related compounds in the more productive libraries, thus, the tendency of related compounds to be present in a given library cannot account for the widely varying hit rate. Although we have not assessed the compound characteristics among libraries in detail, compounds in a given library may have similar physicochemical properties that result in favorable solubility and stability in 5FOA medium or allow uptake by yeast cells. As such, these libraries might serve as a prioritized collection for future yeast growth-based screens.

Eleven of the 30 compounds acquired based on 5FOA-growth results inhibited PDE8A effectively in *in vitro* enzyme assays. Of these compounds, BC8-15 displayed the lowest IC_50_ although it did not produce the highest optical density in the 5FOA assay growth assay. Differences in the relative effectiveness of compounds in the growth assay versus the *in vitro* enzyme assay could reflect differences in the behavior of PDE8A in the *S. pombe* cytoplasm as compared to the environment of an *in vitro* enzyme assay. Alternatively, compounds may appear less effective in the growth assay as compared to the *in vitro* enzyme assay due to relatively poor solubility in the 5FOA growth medium, poor uptake or significant efflux by the *S. pombe* cells, chemical instability during the two-day growth period, or interactions with other *S. pombe* proteins to reduce growth.

**Figure 6:**
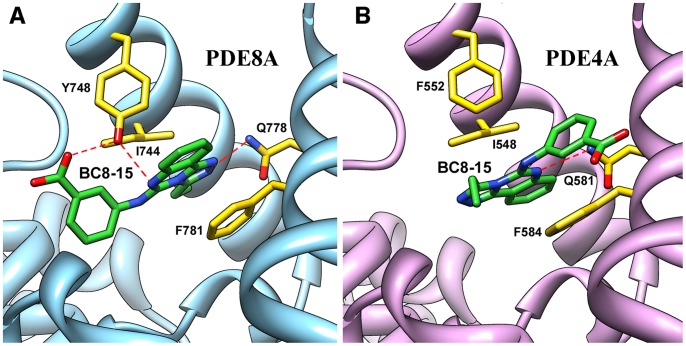
Docking simulation of BC8-15 with PDE8 and PDE4. The binding of BC8-15 with lowest energy was searched by the AutoDock4 software and visualized by UCSF Chimera ^26^. A) Proposed binding orientation of BC8-15 in PDE8A (PDB ID: 3ECN) suggests three hydrogen bonding interactions (shown with dashed lines) through Y748 and Q778. B) BC8-15 adopts a different binding orientation with PDE4A (PDB ID: 2QYK) that favors mostly hydrophobic interactions.

To better understand the pharmacophore of BC8-15 and to identify chemical moieties important for selectivity toward PDE8 and/or PDE4, we tested the inhibitory potential of several BC8-15 derivatives via *in vitro* enzyme assays. Comparing BC8-15 to BC8-15A (Figure 3), we see that replacing the ethyl group on the triazole ring with a methyl group decreases inhibition of both PDE8A and PDE4A. A comparison of activity and structure of BC8-15C versus BC8-15 (Figure 3) reveals a role for the carboxyl group on the phenyl ring (i.e. a benzoic acid) with regard to activity toward PDE8A, but not PDE4A (several other related compounds that lack this carboxyl group displayed little to no activity against PDE8A, but retained activity against PDE4A; data not shown). To begin to understand how BC8-15 binds to PDE4 and PDE8, docking analyses using PDE4A and PDE8A structures were carried out. As shown in Figure 6, BC8-15 appears to be in a different orientation when bound to PDE8A than to PDE4A, although in both orientations BC8-15 is sandwiched between a phenylalanine and isoleucine residue (F781 and I744 in PDE8A and F584 and I548 in PDE4A) through highly possible π-π and CH-π interactions. This model suggests that the carboxylic acid group of BC8-15 interacts with PDE8A through the Y748 residue while this interaction is not involved in its binding to PDE4A (unlike PDE8A and PDE8B, PDE4 and PDE7 enzymes have a phenylalanine at this position rather than a tyrosine), and is consistent with our finding that removal of the carboxylic acid moiety predicted to interact with Y748 from the phenyl ring of BC8-15 reduces its potency as a PDE8 inhibitor, but not as a PDE4 inhibitor (compare BC8-15 and BC8-15C, Figure 3). This tyrosine residue has been previously implicated in inhibitor insensitivity by PDE8A through an unfavorable interaction with IBMX in the PDE8A1-IBMX crystal structure (PDB ID: 3ECN) and by mutation analysis of Y748 to phenylalanine that increases the sensitivity to nonselective PDE inhibitors [31]. The same residue in PDE8B (referred to as Tyr253) was suggested to make a hydrogen bond with a recently-described PDE8B selective inhibitor [32].

In light of the previously-demonstrated roles for PDE8A and PDE8B in steroidogenesis [7], [10], [12], we tested the effect of BC8-15 on steroidogenesis in mouse Leydig cells. In these cells, testosterone production is stimulated by lutenizing hormone (LH) through a cAMP/PKA pathway [33]. Leydig cells from PDE8A knock-out mice are sensitized to LH for testosterone production and further sensitized by IBMX treatment, suggesting the involvement of another PDE [7]. Shimizu-Albergine et al. (2012) subsequently showed that PDE8A, PDE8B, and PDE4 regulate basal steroidogenesis in mouse Leydig cells, which also express PDE1A, PDE1C, PDE2A and PDE3B, as simultaneous inhibition of PDE4 and PDE8 synergistically-elevated basal steroid production [12]. Consistent with these results, we show that 1) BC8-15 elevates steroid production from both primary Leydig cells and MA-10 cells, 2) the amount of testosterone released by PDE8A^−/−/^B^−/−^ knock-out cells is similar when treated with either BC8-15 or rolipram, and 3) the effect of BC8-15, BC8-15A, and BC8-15C on progesterone release by MA-10 cells (Figure 5) corresponds to their activity against PDE8 (Figure 3). As testosterone release by PDE8A^−/−/^B^−/−^ cells is similar upon treatment with either BC8-15 or rolipram, the effect of BC8-15 on wild type cells appears to be solely due to PDE8 and PDE4 inhibition. Finally, while PDE11A is moderately expressed in Leydig cells [34], and both PDE10A and PDE11A transcripts are present in testis [6], [35], inhibition of these PDEs by BC8-15 and its analogs (Figure 3) does not appear to play a significant role in regulating testosterone release by Leydig cells, as suggested by the lack of a biological response to BC8-15C (Figure 5). These results argue that the activity against PDE8 is essential to the biological effect of these compounds, while work by Shimizu-Albergine et al. (2012), showing that PDE4 becomes the predominant cAMP-hydrolyzing PDE in the absence of PDE8, suggests that the PDE4 inhibitory activity of BC8-15 magnifies its biological efficacy, at least in this system [12]. Thus, our studies support the proposal that a combination PDE4/8 inhibitor might be a good therapeutic candidate for treatment of male infertility or for the maintenance of testicular function in patients receiving testosterone therapy due to its ability to stimulate testosterone production [7].

Finally, the commercially-available compounds BC8-15, BC8-15A, and BC8-15C represent a useful chemical toolset for dissecting the biological roles of the PDE4, PDE8, PDE10A, and PDE11A enzymes in tissue culture. Each compound displays a unique profile against these four PDE families and could be used to implicate individual or combinations of PDEs in a biological process of interest.
